# Incidence and farmers’ knowledge of aflatoxin contamination and control in Eastern Democratic Republic of Congo

**DOI:** 10.1002/fsn3.735

**Published:** 2018-07-16

**Authors:** Patchimaporn Udomkun, Tesfamicheal Wossen, Nsharwasi L. Nabahungu, Charity Mutegi, Bernard Vanlauwe, Ranajit Bandyopadhyay

**Affiliations:** ^1^ International Institute of Tropical Agriculture (IITA) Bujumbura Burundi; ^2^ IITA Nairobi Kenya; ^3^ IITA Bukavu Democratic Republic of the Congo; ^4^ IITA Ibadan Nigeria

**Keywords:** aflatoxin contamination, awareness, Farmers, Kendal's concordance, perception

## Abstract

Despite efforts to reduce aflatoxin contamination and associated mycotoxin poisoning, the phenomenon continues to pose a public health threat in food and feed commodity chains. In this study, 300 samples of cassava, maize, and groundnut were collected from farmers’ households in Eastern DRC and analyzed for incidence of aflatoxins. In addition, the farmers’ level of knowledge of the causes and consequences of contamination and the measures for prevention were also examined by administering questionnaires to a cross section of 150 farmers. The results showed the presence of aflatoxins in all samples, with levels ranging from 1.6 to 2,270 μg/kg. In 68% of all samples, total aflatoxin contamination was above 4 μg/kg, the maximum tolerable level set by the European Union. Farmers ranked high humidity, improper storage practices, and poor soils as potential causes of aflatoxin contamination and changes in color, smell, and taste, and difficulty in selling crops as consequences. They identified crop management practices as the most effective way to control contamination. The results also revealed that most farmers apply preharvest crop management practices as a means of controlling contamination. More educated households were more knowledgeable about aflatoxins. Female‐headed and married households were less likely to be willing to pay for aflatoxin control. About 28% of farmers claimed to be willing to allocate resources to seed intervention while a smaller proportion agreed to pay for training and information services. The result further suggests that an adoption of pre‐ and postharvest technologies together with awareness creation is still required to reduce aflatoxin contamination in the country.

## 1 INTRODUCTION

Nutritional security is effective when all people can always consume food of sufficient quantity and quality in terms of variety, diversity, nutrient content, and safety to meet their dietary needs and food preferences for an active and a healthy life (FAO/AGN, 2012). A recent study on the cost of malnutrition reveals that under‐nutrition, micronutrient deficiencies, and obesity currently cost the global economy up to US$3.5 trillion and are a major impediment to global efforts to reduce poverty (Global Panel, 2016; World Bank Group, 2016). In sub‐Saharan Africa (SSA), much of the attention on improving nutrition has focused on reducing under‐nutrition and micronutrient deficiencies. Although some protein‐ and micronutrient‐rich foods have been recommended for consumption, some of them such as legumes and livestock products (milk and cheese) are often the most risky foods as they are also susceptible to contamination by mycotoxins (Sirma et al., 2014).

Aflatoxins among the various mycotoxins have garnered significant attention owing to their negative and even carcinogenic effects on human and animal health. Although they are mainly produced by several *Aspergillus* species, the major causal agent of contamination globally is *Aspergillus flavus* (Klich, 2007). There are four major aflatoxins, B_1_, B_2_, G_1_, and G_2_; however, aflatoxin‐B_1_ is the most toxic and prevalent and is classified as a Group 1 carcinogen by the International Agency for Research on Cancer (IARC, 2002). High‐dose exposure to aflatoxin concentrations can cause acute health effects such as vomiting, abdominal pain, and even death (Probst, Njapau, & Cotty, 2007; Sherif, Salama, & Abdel‐Wahhab, 2009), while sublethal chronic exposure may lead to stunting in children, immune system suppression, and liver cancer (Chan‐Hon‐Tong, Charles, Forhan, Heude, & Sirot, 2013; Wu & Khlangwiset, 2010). In 1981, for instance, the outbreak of aflatoxicosis due to consumption of maize contaminated with 3.2–12 mg/kg of aflatoxin‐B_1_ caused fatalities in Kenya (Obura, 2013). In another severe outbreak, also reported in the Eastern Province of Kenya, contamination was found to be the cause of over 125 deaths in 2004–2005 (Azziz‐Baumgartner et al., 2005). Williams et al. (2004) have indicated that over 5 billion people living in low‐income countries are at risk of chronic exposure to aflatoxins. In animals, aflatoxins may lower resistance to diseases, interrupt vaccine‐induced immunity, and adversely affect growth and reproduction, causing serious economic losses (CAST, 2003; Fink‐Gremmels, 1999).

Aflatoxin contamination is common in low‐income tropical countries such as the Democratic Republic of Congo (DRC), where temperature and relative humidity create an atmosphere favorable for the proliferation of aflatoxigenic fungi (Kamika, Ngbolua, & Tekere, 2016; Kamika & Takoy, 2011). Apart from climatic conditions, socioeconomic factors contribute to aflatoxin contamination, including (a) informal marketing systems, (b) inadequate transportation modes, (c) unavailability of needed materials, tools, and equipment, (d) lack of information and knowledge on appropriate pre‐ and postharvest management, and (e) poor governmental regulations and legislation. Moreover, DRC has experienced conflicts, resulting in poor outcomes in health, education, and living standards. Food insecurity and malnutrition are also a common occurrence, especially among children, in resource‐poor households. The interplay between safety of food and adequacy of food is therefore crucial when addressing the aflatoxin problem in the country.

Although aflatoxin contamination is known to be common in low‐income countries, there is little if any documentary evidence, so the impact of eating contaminated food in SSA is underestimated (Grace et al., 2015). Moreover, key factors, such as farmers that could play a significant role in control, have limited knowledge about the causes of aflatoxin contamination, its effects, and measures of control. In effect, they are not willing to incur the costs of controlling aflatoxin contamination especially as most of the transactions are in informal markets without strong regulations (Sirma et al., 2014). This study tries to address four questions regarding aflatoxins in Eastern DRC: (a) What is their incidence in crop products from farmers’ households? (b) What are the correlates of the degree or the extent of aflatoxin contamination? (c) Are farmers knowledgeable about the causes and effects of aflatoxin contamination and ways of control? (d) Are farmers willing to pay for control measures? Information generated by this study will provide guidance for interventions required for aflatoxin mitigation in staple crops in DRC.

## 2 MATERIALS AND METHODS

### 2.1 Sample collections

**Figure 1:**
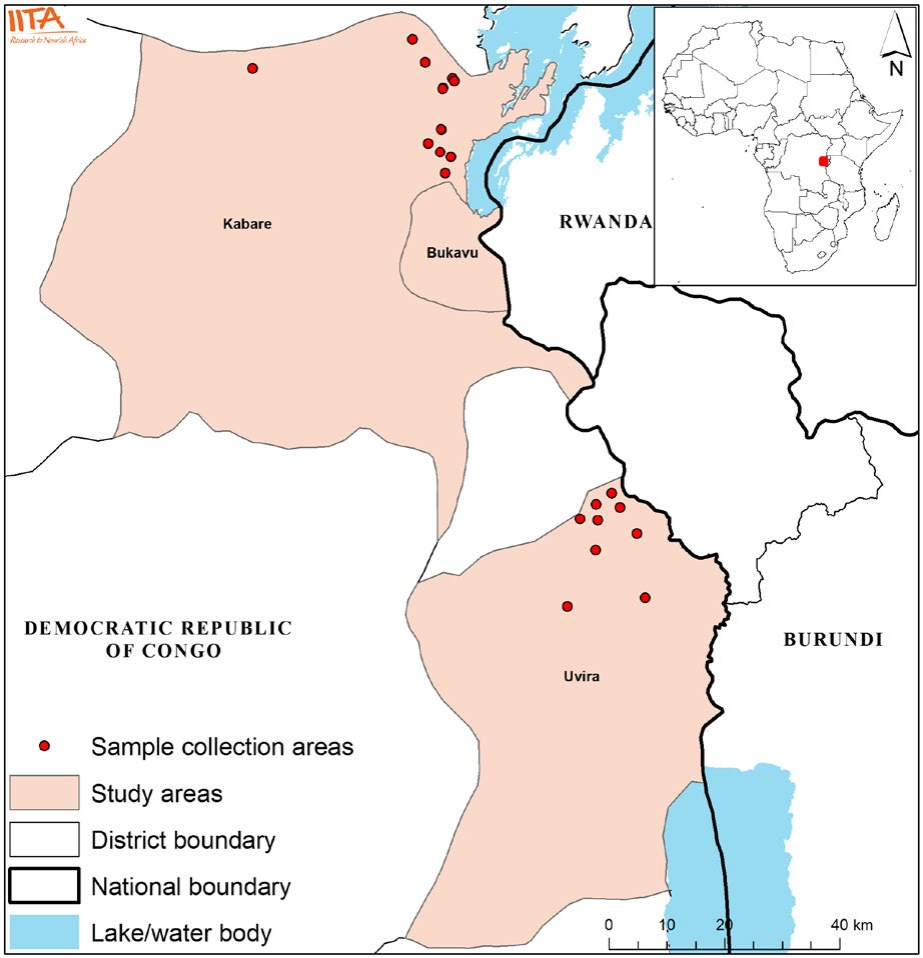
Sampling areas in Eastern Democratic Republic of Congo

Data were collected during May and June 2017 in specific areas of Kabare and Uvira districts in Eastern DRC. These areas were contrasted by altitude, average temperature, and rainfall. Kabare is considered as high altitude area (1,463–2,000 m above sea level) with an average temperature of 20°C and annual rainfall >1,000 mm, while Uvira represents low altitude area (1,000–1,200 m above sea level) with an average temperature of 26°C and annual rainfall 800–1,000 mm. The farm households in these areas are mainly subsistence farmers and grow rainfed crops. The main food crops in Kabare district include cereal crops such as maize and legumes, while in Uvira district the main crops are cassava and legumes. The two districts were selected as they are the major production areas of maize, cassava, and groundnuts. Due to security reasons, the study was conducted in the north‐eastern part of Kabare and northern part of Uvira. To collect a representative data set, we first obtained the list of villages in each district from Provincial Inspection for Agriculture and Livestock (IPAPEL). From each district, 10 villages were then randomly selected. These include Nshebeyi, Cituzo, Konge, Miti centre, Cibinda, Buhandahanda, Kashenyi, Cirheja, Mwanda, and Kahungu villages in Kabare district and Lupango, Katagota, Ndolera, Nyamutire, Luvungi, Muturule, Lubarika, Sange, Kiliba, and Kamanyola villages in the Uvira district. From each selected village, households were selected for interview using probability proportional to size (PPS) sampling approach. As the sampling takes into account the population size of the village, the number of interviewed households per village is not the same. In particular, more households were selected in villages with more population size for interview. In total, 150 farm households were selected for this study. The geographical attribute of the sampling areas is shown in Figure 1.

As maize, groundnut, and cassava are the main staple crops, a total of 300 food samples, consisting of 30 dried cassava roots, 30 cassava flours, 50 maize grains, 50 maize flours, 50 groundnut grains, 50 roasted groundnuts, and 40 groundnut flours, were collected from 150 farm households. The samples were collected during visits for interview. Two samples were collected from each individual household, depending on available samples in each household.

All samples were produced by farmers and used for household consumption. For each sample, 0.5–1.0 kg of the commodity was collected from different parts of the container and thoroughly mixed. Samples were labeled with the name of the farmer, household number, village, and collection date, and then subdivided into two portions. The first was kept as a back‐up; the second was examined to determine the level of aflatoxin contamination. All samples were dried to 9%–11% moisture content, sealed in polyethylene bags under normal atmospheric conditions. The package seal was inspected frequently to avoid any possibility of fungal proliferation, insect infestation, and leakage. Subsequently the sealed packages were stored at a temperature of 4°C without direct sunlight until further analysis.

### 2.2 Chemical analysis

#### 2.2.1 Analysis of total aflatoxins

For each sample except flour, 200 g was ground into fine powder using a laboratory blender (model 37BL85; Dynamics Corporation of America, USA). Approximately 10 g of the ground sample was added to 50 ml 65% ethanol (v/v) in a 100‐ml media bottle. The resulting suspension was shaken (model HS 501 D Shaker; IKA, Germany) at 200 rpm for 3 min to extract aflatoxins. The suspension was allowed to settle, then filtered through Whatman No. 1 paper and the filtrate collected.

To analyze the aflatoxin concentration, a Reveal Q+ test kit (Neogen Corporation, USA) was used as a single step lateral flow immuno‐chromatographic assay based on a competitive immunoassay format. A total of 500 μl of diluent were mixed with 100 μl of the sample filtrate and then carefully mixed by being pipetted up and down five times in a dilution cup. A 100 μl portion of the mixture was transferred to a clean sample cup. Subsequently, a Reveal Q+ for aflatoxin test strip was placed into the sample cup for 6 min, removed, and inserted in the AccuScan^®^ reader (AccuScan Pro, model AX‐2; Neogen Corporation, Australia). Aflatoxin concentration was displayed in parts per billion (ppb; μg/kg). All samples were analyzed in duplicate.

#### 2.2.2 Validation of total aflatoxins

The standard of total aflatoxins was purchased from Sigma‐Aldrich Chemie GmbH (Taufkirchen, Germany). To test the sensitivity of the method, the standard solution at two different concentrations was added to all samples. The extraction and recovery of the spiked samples were performed in duplicate as previously described. The validation of Reveal Q+ method was carried out with the determination of the recoveries and the coefficient of variation (%CV) as presented in Table 1.

**Table 1:** Validation data of methods for total aflatoxins

Category	Total aflatoxin level added (μg/kg)	Recovery (%)	Coefficient of variation (%CV)
Cassava			
Dried root	2.0	80.7	3.2
	10.0	81.4	3.5
Flour	2.0	86.2	2.7
	10.0	85.6	3.8
Maize			
Grain	2.0	91.4	2.2
	10.0	91.8	3.6
Flour	2.0	87.5	3.8
	10.0	89.6	4.4
Groundnut			
Grain	2.0	91.2	2.3
	10.0	90.6	1.8
Roasted	2.0	89.7	1.9
	10.0	85.4	2.3
Flour	2.0	88.8	2.0
	10.0	86.7	3.1

### 2.3 Survey design

After selection of 150 respondents who had the main responsibility for their household's farm activity, interviews were conducted using semi‐structured questionnaires designed in English and translated into French. It was pretested and administered by trained enumerators. Each interview began with the enumerators confirming the respondent was the person responsible for the survey and explaining the aims of the study. Where the farmers scheduled for interview were not at home, the enumerators rescheduled the interviews.

To characterize the socio‐demographic backgrounds of respondents, data on the following variables were collected: relationship with household head, household size, sex, age, marital status, education, off‐farm activities, and household income and sources. As the word “aflatoxin” is technical, its meaning was explained to farmers in their local languages (Swahili and Mashi). Thereafter, farmers were prescreened, and only those who indicated that they knew aflatoxins were asked to evaluate their knowledge and awareness about the causes, consequences of aflatoxin contamination, and preventive measures. The survey instrument included a detailed list of questions such as “are aflatoxins caused by fungi?” to measure farmers knowledge about aflatoxin. Similarly, questions such as “are you aware of aflatoxin contamination in crops in the field and during storage?” were asked to assess farmers awareness about aflatoxin. Moreover, only farmers who knew aflatoxins were asked to rank their perception of causes and consequences and knowledge of preventive measures on a five‐point scale (strongly agree = 1, fairly agree = 2, neutral = 3, a little agree= 4, and not at all = 5). Lastly, the willingness to pay for a package of interventions was examined using the following question: “are you willing to pay for a package of interventions to address aflatoxin contamination?” Information was collected by asking farmers to directly report their willingness to pay as an investment.

### 2.4 Data analysis

The data for aflatoxin contamination in foods were statistically analyzed using SAS (Ver. 9.4; SAS Institute Inc., Cary, NC, USA). Analysis of farmers’ knowledge, awareness, and willingness to pay was performed using STATA (version 14.0; StataCorp, TX, USA). To describe the responses, basic statistics such as means and frequencies were computed. Chi‐square (χ^2^) and *t* tests were used to examine differences in the responses from farmers. They were made to rank their perceptions about the cause and consequence of aflatoxin contamination, and preventive measures using the five‐point Likert Scale. The mean scores of each of the ranked factors were computed to identify the most important.

Kendall's coefficient of concordance (*W*) was computed to measure agreement in the ranks of the factors (*m*) by the farmers (*N*) (Siegel & Castellan, 1988). Kendall's rank correlation coefficient was calculated as follows:

(1) $$W=\frac{S}{\frac{1}{2}K^{2}\left(N^{3}-N\right)}$$

where *K* is the number of ranking criteria and *S* is the sum of squares of deviation from rank means. Kendall's rank correlation provides a distribution‐free test of independence and measures the strength of dependence between two variables being compared to judge the significance of the computed coefficient using a chi‐square test. A larger coefficient signifies strong agreement.

To better understand the association between household farmer characteristics and the level of aflatoxin contamination, we also estimated the main determinants of aflatoxin contamination using a regression model. In particular, let *Y*_i_ be the level of aflatoxin concentration found in the food items consumed by a particular farmer and let *X* be a vector of socioeconomic factors such as education, income, age, etc., that affect aflatoxin concentration level. The full list of the main determinants is presented in Table 2. The empirical relationship between aflatoxin concentration and the above socioeconomic variables is then specified as follows:

(2) $$Y_{i}=\alpha_{0}+\vartheta X_{i}+u_{i}$$

where α_0_ is the intercept term and *ϑ* is the error term. The above specification was estimated using Ordinary Least Square (OLS). Further, to explore the main determinants of farmers’ knowledge, awareness, and willingness to pay about aflatoxin, the following Probit model specification was estimated:

**Table 2:** Description of variables used in the Probit model

Variable	Description
Household size	Number of members in a household
Sex	1 if female, 0 otherwise
Age	years
Marital status	1 if married, 0 otherwise
Education level	1 if educated, 0 otherwise
Annual income	Household income for last 12 months (USD)

(3) $$W_{i}=\alpha_{0}+\gamma X_{i}+\varepsilon_{i}$$

*W*_i_ represents one of the three outcome variables: (a) a dummy variable that takes on a value of one if a farmer is knowledgeable about aflatoxin and zero otherwise, (b) a dummy variable that takes on a value of one is a farmer has awareness about aflatoxin and zero otherwise, and (c) a dummy variable that takes on a value of one if a farmer is willing to pay for aflatoxin control measures and zero otherwise. The knowledge level of farmers was measured using specific questions such as (a) aflatoxin is caused by fungi (Yes/No), (b) aflatoxin cause cancer in humans (Yes/No), (c) aflatoxins delay child growth (Yes/No), and (d) aflatoxin contamination causes fever in animals (Yes/No). In the Probit model, where we examined the determinants of knowledge, we created a dummy variable that takes on a value of one if the household responds “yes” to at least one of the above questions and a value of zero, otherwise.

Similarly, awareness level of farmers about aflatoxin contamination and causes was measured using the responses for the following questions: (a) I am aware of aflatoxin contamination in crops in the field and during storage (Yes/No), (b) I am aware of aflatoxins in foods on the table after harvest (Yes/No), (c) I am aware of aflatoxins in milk and dairy products (Yes/No), (d) I am aware of the harmful effects of aflatoxins on humans (Yes/No), and (e) I am aware the effects of aflatoxins on animals (Yes/No). Awareness was also measured by a dummy variable that takes on a value of one if the household responds “yes” to at least one of the above questions and a value of zero, otherwise. The detailed questions used to construct knowledge and awareness are presented in Table 3. Finally, willingness to pay was measured using the following simple hypothetical question “are you willing to pay for intervention to address aflatoxin contamination?” (Yes/No). Based on the response, we constructed a dummy variable which takes on a value of one if the answer for the above question is “yes” and zero, otherwise. For each outcome (knowledge, awareness, and willingness to pay), a separate Probit model was estimated. As before, *X* denotes a vector of socioeconomic variables as given in Table 2 and γ is a vector of parameters to be estimated.

**Table 3:** Extent of knowledge and awareness of farmers who knew about aflatoxins in Eastern Democratic Republic of Congo on aflatoxin contamination

Factors	Response (*N *=* *127)
1. Occurrence of aflatoxins	
1.1 Aflatoxins can be present in crops	108 (84.7)^a^
1.2 Aflatoxin contamination occurs at any time of plant growth^b^	104 (96.0)
1.3 Aflatoxins can be transferred to animals	64 (50.0)
1.4 Aflatoxins can be transferred into milk and dairy products	69 (54.0)
1.5 Aflatoxins can be transferred into breast milk	80 (63.3)
1.6 I am aware of aflatoxin contamination in crops in the field and during storage	38 (30.0)
1.7 I am aware of aflatoxins in foods on the table after harvest	39 (30.7)
1.8 I am aware of aflatoxins in milk and dairy products	46 (36.0)
2. Cause of aflatoxin contamination	
2.1 Aflatoxins are caused by fungi	75 (59.3)
2.2 High levels of rain during harvesting	102 (80.7)
2.3 Delayed harvesting	80 (63.3)
2.4 Delayed drying	94 (74.0)
2.5 Insect infestation causes	97 (76.0)
2.6 Broken and bruised crops increase a chance of contaminations	90 (70.7)
2.7 Crops which contain foreign materials promote aflatoxins	80 (63.3)
2.8 Poor storage conditions promote aflatoxin contamination in crops	107 (84.0)
3. Effect of aflatoxin contaminations	
3.1 Fungi produce toxic compounds	95 (74.7)
3.2 Crops that differ in taste promote aflatoxins	85 (66.7)
3.3 Crops that are discolored produce aflatoxins	100 (78.7)
3.4 Aflatoxin contamination reduces animal productivity	66 (52.0)
3.5 Aflatoxin contamination causes stunting in animals	12 (9.3)
3.6 Aflatoxin contamination causes fever in animals	0 (0.0)
3.7 Aflatoxin contamination cause death in animals	0 (0.0)
3.8 I am aware of the harmful effects of aflatoxins on humans	62 (48.7)
3.9 I am aware the effects of aflatoxins on animals	69 (54.0)
3.10 Some liver diseases have been linked to intake of aflatoxins	93 (73.3)
3.11 Aflatoxins cause cancer in humans	90 (70.7)
3.12 Aflatoxins delay child growth	102 (80.7)
3.13 Aflatoxin contamination can reduce the price of crops	106 (83.3)
3.14 Aflatoxin‐contaminated food cannot be exported to some countries	82 (64.7)

a Number of occurrence (percentage occurrence).

b *N *=* *108 (only the farmers who can answer question 1.1 were then asked question 1.2).

## 3 RESULTS

### 3.1 Occurrence of aflatoxins in crop samples

The occurrence and concentration of total aflatoxins in crop samples collected from farmers’ household in Eastern DRC are summarized in Table 4. All 300 samples were contaminated with aflatoxins that ranged from 1.6 to 2,270 μg/kg. As DRC does not have regulations for aflatoxins, we applied the EU standard for maize as a comparison for all crop samples. About 68% of total samples contained aflatoxins above the EU's 4 μg/kg permissible limit for total aflatoxins in maize intended for human consumption (EU, 2007, 2010). In cassava samples, aflatoxin levels ranged from 1.6 to 5 μg/kg. More than 85% of the samples met the EU regulatory threshold for aflatoxins, and all the samples met the proposed East African regulatory threshold of 10 μg/kg. Aflatoxin levels in maize samples ranged from 2.5 to 325 μg/kg. About 66% of the maize grain samples and 88% of the maize flour samples did not meet the EU regulatory threshold. In groundnut samples, total aflatoxin concentration ranged from 2.7 to 2,270 μg/kg. The highest level was found in groundnut flour (2,270 μg/kg), followed by roasted groundnut (865 μg/kg), and dried kernels (25 μg/kg). About 68% of the groundnut samples exceeded EU aflatoxin regulatory limits. None of the groundnut flour samples were fit for human consumption according to any existing global regulation. In addition, aflatoxins were found in processed groundnut more than in unprocessed, dried grain.

**Table 4:** Distribution and level^b^ of total aflatoxins in samples found in farmers’ households of Eastern DRC

Category	Distribution of total aflatoxins (*N *=* *300)			Level of total aflatoxins^b^		
	Incidence^a^	Average (μg/kg)	Range (μg/kg)	<4 μg/kg	4–10 μg/kg	>10 μg/kg
Cassava						
Dried root	30/30	3.5	2.6–5	24 (80)^c^	6 (20)	—
Flour	30/30	2.8	1.6–4.8	27 (90)	3 (10)	—
Maize						
Grain	50/50	25.0	2.7–320	17 (34)	19 (38)	14 (28)
Flour	50/50	32.5	2.5–325	6 (12)	19 (38)	25 (50)
Groundnut						
Grain	50/50	7.1	2.7–25.1	14 (28)	24 (48)	12 (24)
Roasted	50/50	76.7	3.2–865	8 (16)	22 (44)	20 (40)
Flour	40/40	495.3	14.1–2,270	—	—	40 (100)
Total	300/300	90.2	1.6–2,270	96 (32)	93 (31)	111 (37)

a Incidence number is represented by the number of positive samples/total sample in a category.

b EU permissible level for total aflatoxins is 4 μg/kg and WHO advisory level is 10 μg/kg for foods intended for direct human consumption.

c The first integer is the number and the integer in parentheses is the percentage of samples containing a specified level of aflatoxins.

### 3.2 Farmer characteristics

The socio‐demographic characteristics of 150 farmers interviewed for this study is presented in Table 5. About 59% were household heads, and the average household size was eight persons. Farmers were of middle age with an average of about 43 years. Over 88% were married, giving an indication of the importance of the marriage institution in the study area. About 75% had completed at least primary education, indicating a measure of literacy. More than 80% were engaged in off‐farm activities that provided alternative sources of income and served as an insurance against shocks. The average household annual income was US$914. Relating this to household size results in an average per capita income of about US$114. The main sources of annual income include harvested produce (72%), permanent employment (24%), and processing (3%).

**Table 5:** Characteristics of farmers interviewed in Eastern Democratic Republic of Congo

Characteristics	Response (*N *=* *150)
Relationship with the household head^a^	
Head	87 (58.8%)
Spouse	58 (39.2%)
Child	3 (2.0%)
Household size (number)^b^	8.37 (3.23)
Sex^a^	
Male	81 (54.4%)
Female	68 (45.6%)
Age (years)^b^	43.3 (13.9)
Marital status^a^	
Single	17 (11.6%)
Married	130 (88.4%)
Education^a^	
None	35 (24.8%)
Basic (primary/junior high)	48 (34.0%)
Secondary (senior high)	51 (36.2%)
Tertiary (college/university)	7 (5.0%)
Off‐farm activities^a^	126 (84.0%)
Annual income (US$)^b^	914 (112.5)
Main sources of income^a^	
Artisan	2 (1.4%)
Permanent employment	35 (23.6%)
Part‐time work	0 (0.0%)
Pension	0 (0.0%)
Harvested produce	109 (72.3%)
Processing	4 (2.8%)

a Number of occurrence (percentage occurrence).

b Mean (standard deviation).

### 3.3 Correlation between household characteristics and aflatoxin occurrence

Results reported in Table 6 indicate a positive and significant correlation between household size and the level of aflatoxin contamination, while a negative and significant correlation was observed between education, household annual income, and level of aflatoxin contamination (Table 6). This result suggests that food samples from larger households are more likely to be contaminated by aflatoxin than samples from smaller households. Food samples from households where the household head had received education and had a high income are likely to have lower levels of aflatoxin contamination compared to households where the household head has little education and that are poor.

**Table 6:** Ordinary least squares (OLS) regression results for aflatoxin contamination

Dependent variable	Level of aflatoxin contamination
Household size	0.180** (0.083)^a^
Sex	0.015 (0.298)
Age	0.007 (0.014)
Marital status	−0.396 (0.626)
Education level	−0.842* (0.499)
Annual income	−0.0001* (0.00006)
Pseudo *R* ^2^	0.104
Correctly classified	76.1%
*N*	150

^a^Robust standard errors are reported in parentheses. ***p <* *0.01, **p <* *0.05, *p <* *0.1.

### 3.4 Perceptions of aflatoxin contamination

Of the 150 farmers, 127 (85%) farmers knew about aflatoxin. Of these 127 farmers, 85% (108 farmers) knew that aflatoxins can be present in crops. Among those who knew aflatoxins can be present in crops (108 farmers), 96% of them (104) farmers agree that infection occurs at any time of plant growth. About 54% knew about the bio‐transfer of aflatoxins to livestock products. Interestingly, 41% did not know that aflatoxins are caused by fungi. This suggests that farmers knew of the occurrence but not of the organism that causes contamination. Regarding the effects of contamination, an average of 73% identified changes in taste and color as the main effect on crops—even when the presence of aflatoxins cannot be detected visually. More than 50% had sufficient perception on the negative impacts of contamination on animal production. Although 80% knew that contamination can reduce the price of crops, the majority were not aware of the risk to human health from contamination.

The farmers who knew about aflatoxins were then asked to rank their perceptions on causes, consequences, and preventive measures of aflatoxin contamination. The results showed that farmers perceived abiotic factors to be the cause of the high prevalence and severity of contamination (Table 7). High humidity was also rated as a cause of very severe contamination. Other causes were rated as moderately severe and included poor soils, poor storage practices, drought stress, contaminated seeds, and delayed harvesting. The farmers’ ignorance of the biotic causes of contamination might be due to their lack of knowledge on the connection between microorganisms and contamination. When asked how they could control contamination, the farmers identified tackling poor storage practices, followed by poor soils, insect infestation, poor field management, and delayed harvest.

**Table 7:** Mean ranking^a^ of farmers’ perception on causes of aflatoxin contamination

Causes	Prevalence of the causes of aflatoxin contamination	Severity of the causes of aflatoxin contamination	Ease of controlling the causes
Biotic			
Microbial infection	2.8 (0.2)^b^	2.8 (0.1)	3.4 (0.3)
Insect infestation	2.5 (0.1)	2.8 (0.2)	2.0 (0.1)
Grazing animals	2.8 (0.3)	3.2 (0.2)	3.4 (0.2)
Rodents	2.5 (0.2)	2.5 (0.1)	2.7 (0.1)
Abiotic			
High humidity	1.0 (0.1)	1.0 (0.0)	2.6 (0.0)
High temperature	1.7 (0.2)	2.3 (0.1)	2.3 (0.1)
Poor soils	1.4 (0.1)	1.8 (0.1)	1.9 (0.1)
Drought stress	1.9 (0.1)	2.3 (0.2)	2.3 (0.0)
Management			
Contaminated seeds	2.4 (0.3)	2.4 (0.1)	2.7 (0.1)
Poor field management	2.0 (0.2)	2.1 (0.2)	2.2 (0.1)
Delayed harvest	1.9 (0.1)	2.4 (0.1)	2.2 (0.1)
Poor storage practices	1.4 (0.0)	1.8 (0.0)	1.4 (0.0)
Kendal's correlation coefficient	0.02	0.02	0.18
Chi‐square	39.0	40.4	404.0
*p*‐Value	0.01	0.01	0.00
*N*	127		

a A five‐point scale ranking (1 =  strongly agree, 2 =  fairly agree, 3 =  neutral, 4 =  a little agree, and 5 =  not at all).

b Mean (standard deviation).

The mean ranking of farmers’ perceptions on the consequences of aflatoxin contamination was calculated (Table 8). The farmers ranked changes in color as the most important consequence, followed by changes in smell and taste, and difficulty in selling crops. These factors are interlinked as they make the product unattractive to consumers and affect farmers ability to sell it, resulting in financial losses. Generally, farmers who knew about aflatoxins were very much aware of the potential ill effects of the consumption of contaminated foods. They also identified delayed child growth as the most severe consequence of contamination, followed by development of liver cancer, change in smell, and difficulty in selling crops, in that order. The ranking was the same for the need for immediate control.

**Table 8:** Mean ranking^a^ of farmers’ perceptions on consequences of aflatoxin contamination

Consequence	Ease of identification of the consequence	Severity of the consequence	Need for control of the consequence
Food and feed			
Change in taste	1.8 (0.1)^b^	2.3 (0.1)	1.9 (0.1)
Change in smell	1.7 (0.1)	1.9 (0.0)	2.0 (0.0)
Change in color	1.4 (0.0)	2.2 (0.2)	2.3 (0.2)
Health			
Development of liver cancer	2.3 (0.2)	1.9 (0.1)	1.7 (0.0)
Delay of child growth	2.1 (0.1)	1.7 (0.1)	1.5 (0.1)
Lower resistance to diseases of animals	2.3 (0.1)	2.2 (0.0)	2.2 (0.2)
Economic			
Difficulty in selling crops	1.7 (0.0)	2.0 (0.1)	1.9 (0.1)
Reduction in marketable price	2.3 (0.1)	2.3 (0.2)	2.2 (0.1)
Kendal's correlation coefficient	0.01	0.03	0.02
Chi‐square	15.3	49.7	33.8
*p*‐Value	0.03	0.00	0.00
*N*	127		

a A five‐point scale ranking (1 =  strongly agree, 2 =  fairly agree, 3 =  neutral, 4 =  a little agree, and 5 =  not at all).

b Mean (standard deviation).

### 3.5 Perceptions of aflatoxin prevention and control

Although farmers were less knowledgeable about the negative impact of aflatoxin contamination on human and animal health, at least 73% knew the importance of good farming practices for prevention (Table 9). The farmers also agreed on the importance of selection of healthy seeds (68%) and use of pest and disease control (67%). They, however, do not appreciate the potential of postharvest management practices for minimizing contamination in agricultural products. To eliminate the risks of fungal development and subsequent aflatoxin contamination, only 12% indicated the use of proper drying as an effective method, while less than 5% understood the importance of proper storage, food processing, and hygienic methods of feeding animals.

**Table 9:** Perception of farmers in Eastern Democratic Republic of Congo on aflatoxin prevention and control measures

Measures	Response (*N *=* *127)
Preharvest management	
Selection of healthy seeds	86 (68.0%)^a^
Good farming practices	93 (73.3%)
Pest and disease control	85 (67.3%)
Postharvest management	
Proper storage	3 (2.7%)
Proper drying	15 (12.0%)
Food processing	7 (5.3%)
Feeding animals with clean seeds	2 (1.3%)

a Number of occurrence (percentage occurrence).

The mean ranking of farmers’ perceptions on measures to prevent aflatoxin contamination is shown in Table 10. It appears that they are likely to rate preharvest interventions as easier preventive methods, particularly the use of resistant varieties, selection of healthy seeds, and seed treatments with chemical fungicide. Most farmers in Eastern DRC believed that these preharvest interventions with biological control are more effective for controlling aflatoxin contamination. For postharvest management, cleaning crops before storage and using anti‐microbial agents were ranked as efficient methods.

**Table 10:** Mean ranking^a^ of farmers’ perceptions on preventive measures for aflatoxin contamination

Causes	Ease of application	Cost	Effectiveness
Preharvest			
Removal of stubble from previous crops	1.8 (0.1)^b^	2.3 (0.1)	2.1 (0.2)
Using resistant varieties	1.4 (0.0)	1.7 (0.0)	1.6 (0.0)
Selection of healthy seeds	1.4 (0.0)	2.1 (0.1)	1.6 (0.1)
Seed treatments with biological control	2.1 (0.2)	2.7 (0.2)	1.9 (0.1)
Seed treatments with chemical fungicide	1.4 (0.1)	2.0 (0.1)	2.0 (0.2)
Using crop rotation	2.0 (0.1)	2.4 (0.1)	2.1 (0.1)
Providing supplement irrigation	2.4 (0.2)	2.9 (0.2)	2.7 (0.2)
Control of pests and diseases	1.8 (0.0)	1.7 (0.0)	1.9 (0.0)
Postharvest			
Avoiding mechanical damage during harvesting	1.9 (0.1)	2.7 (0.1)	2.2 (0.2)
Cleaning crops before storage	2.0 (0.1)	1.8 (0.1)	1.8 (0.1)
Fumigation of storage room	2.7 (0.2)	2.7 (0.3)	2.5 (0.2)
Using anti‐microbial agents	2.1 (0.1)	2.0 (0.2)	2.1 (0.1)
Kendal's correlation coefficient	0.00	0.02	0.01
Chi‐square	8.1	19.6	11.0
*p*‐Value	0.07	0.05	0.45
*N*	127		

a A five‐point scale ranking (1 =  strongly agree, 2 =  fairly agree, 3 =  neutral, 4 =  a little agree, and 5 =  not at all).

b Mean (standard deviation).

### 3.6 Farmers willingness to pay for aflatoxin control measures

Table 11 presents farmers self‐reported willingness to pay for some of the aflatoxin control measures. The result suggests that farmers are willing to pay for some of the control interventions such as training (73%), seeds of resistant varieties (83%), seed treatment (75%), and information (51%) that can reduce contamination. Without any clear demand for aflatoxin‐free crops in these countries, farmers claimed to be willing to allocate more of their resources (32%) to improved seeds as opposed to training (27%) and information (20%). Nonetheless, the lower levels of investment perhaps reflect poor awareness of the hazards of contamination.

**Table 11:** Willingness of farmers to pay for aflatoxin control interventions in Eastern Democratic Republic of Congo

Interventions	Responses (*N *=* *127)
Willingness to pay^a^	
Any intervention	127 (100.0%)
Training	92 (72.5%)
Information	65 (50.8%)
Seeds of resistant varieties	105 (82.5%)
Seed treatment	96 (75.4%)
Percentage of investment to be allocated^b^	
Any intervention	27.0 (5.2)
Training	27.0 (4.8)
Information	20.3 (6.4)
Seeds of resistant varieties	31.8 (2.5)
Seed treatment	25.7 (3.3)

a Number of occurrence (percentage occurrence).

b Mean (standard deviation).

### 3.7 Factors influencing farmers’ knowledge, awareness, and willingness to pay

The main determinants of farmers awareness and knowledge about aflatoxin contamination as well as their willingness to pay for aflatoxin control measures are presented in Table 12. Results of the Probit model reveal that large households were observed to be less knowledgeable and aware about aflatoxin contamination compared to small households. Female‐headed households were also found to be less willing to pay for aflatoxin control measures compared to male‐headed households. Further, we found a negative and significant association between marital status and willingness to pay for aflatoxin control measures. This indicated that single as well as separated or divorced farmers were more willing to pay for control than those who were married. Education was significant and positively correlated with higher levels of knowledge concerning the occurrence, cause, and effect of aflatoxin contamination. Annual income, on the other hand, only influenced farmers knowledge and awareness about aflatoxin contamination but not willingness to pay.

**Table 12:** Probit model estimates of the determinants of farmers’ knowledge and awareness of aflatoxin contamination and their willingness to pay for control

	Knowledge	Awareness	Willingness to pay
Household size	−0.116** (0.050)	−0.101** (0.047)	−0.061 (0.047)
Sex	0.206 (0.305)	0.156 (0.288)	−0.520* (0.284)
Age	0.019 (0.012)	0.011 (0.011)	−0.009 (0.011)
Marital status	0.220 (0.465)	0.337 (0.399)	−1.287** (0.543)
Education level	0.928** (0.378)	0.534 (0.327)	−0.070 (0.354)
Annual income	0.252* (0.131)	0.344*** (0.115)	−0.021 (0.118)
Pseudo *R* ^2^	0.104	0.080	0.086
Correctly classified	76.1%	75.2%	74.3%
*N*	113	113	113

*Note*

Robust standard errors are reported in parentheses. ***p <* *0.01, **p <* *0.05, *p <* *0.1.

## 4 DISCUSSION

The results from samples collected from farmers’ households suggest that fresh cassava is safe from aflatoxin contamination; however, processing methods such as heat treatment, sun drying, and freezing may alter the ability of cassava to block toxin production, leading to secondary contamination (Abass, Awoyale, Sulyok, & Alamu, 2017). Another possible explanation for this observation is that the effect of the fermentation process generally employed in processing roots to dried cassava and cassava flour favors the growth of lactic acid bacteria (LAB) or some microorganisms such as *Saccharomyces cerevisiae* strains. The ability of these microorganisms to bind or degrade aflatoxins, especially aflatoxin‐B_1_ and aflatoxin‐M_1_, in foods and feeds has been reported (El‐Nezami & Gratz, 2011; Peltonen, El‐Nezami, Haskard, Ahokas, & Salminen, 2001; Shetty, Hald, & Jespersen, 2007).

In maize samples, our findings support those of Kamika et al. (2016), who reported that aflatoxin contamination in the DRC occurred along the maize supply chain, with a drastic increase of up to 500 times from preharvest (3.1–103.9 μg/kg) to city stores (2,070.5 μg/kg), and to distribution markets (2,806.5 μg/kg). They attributed this trend to inappropriate storage practices as well as a lack of drying facilities in the country. The higher levels of aflatoxin contamination in processed groundnut could be explained by the fact that processed groundnut, often prepared from low quality materials, can be exposed to a wide range of environmental conditions, such as high temperature and humidity as well as oxygen and mold, which can trigger further increases in contamination. Moreover, contamination usually increases during storage, thus as samples were taken after undergoing some storage time in markets, the values obtained were likely to be higher than others obtained before storage. Nonetheless, other factors including biological, nutritional, and climatic factors can be responsible for aflatoxin contamination, especially in groundnut and maize, some of which are either difficult or impracticable to control (Bankole, Schollenberger, & Drochner, 2006; Ezekiel et al., 2013; Monyo et al., 2012).

We found a negative and significant correlation between the level of aflatoxin concentration and the level of education and income of farmers. This result suggests that more educated and wealthier farmers tend to be more conscious about the quality of the food they consume as aflatoxin concentration in their foods is much lower compared to the concentration found in the foods of other farmers in the study area. It also appears that educated farmers who practiced effective crop husbandry had a lower level of aflatoxin contamination in products than farmers with little education. Regarding income, Robert, Manolis, and Tanner (2003) stated that lower income consumers are more concerned about value for money and meeting their basic needs (Erasmus, Donoghue, & Dobbelstein, 2014).

For aflatoxin prevention and control, most farmers mentioned high costs, unavailability of technology, and low awareness of potential benefits as the main reasons for not applying postharvest management. This corroborates with the finding of Marechera and Ndwiga (2014) who reported the low use of modern postharvest aflatoxin control technologies in Eastern Kenya. Considering the cost associated with control methods, most farmers indicated that the use of resistant varieties and control of pests and diseases are the most expensive. This was followed by cleaning crops before storage, seed treatment with chemical fungicide, and using anti‐microbial agents. Midega, Murage, Pittchar, and Khan (2016) reported that the low use of chemical applications is due to lack of information on appropriate and effective products as well as the inability to afford these chemicals. Kumar and Popat (2010) noted that farmers were indifferent to aflatoxin contamination due to many factors such as their perceptions of aflatoxins as an economic constraint, low levels of awareness and knowledge, and market restrictions. The priority for farmers in these countries was increasing crop productivity as opposed to increasing quality. In addition, the lack of regulatory enforcement or even a definition of acceptable limits does not affect the markets; hence, farmers simply do not bother to control aflatoxin contamination.

When examining factors influencing farmers’ knowledge and awareness about aflatoxin contamination, we found a negative and statistically significant correlation between household size and knowledge and awareness. This may be due to the high consumption pressure (to fulfill family consumption needs) associated with a large family, limiting their ability to learn about the quality of the food they consume. Many studies indicated that women had a greater knowledge of fungal and aflatoxin contamination compared to men (Sabran, Jamaluddin, Abdul Mutalib, and Abdul Rahman (2012); Saulo & Moskowitz, 2011). In contrast, we found that respondents in female‐headed households were less willing to pay for aflatoxin control. Jolly, Bayard, Awuah, Fialor, and Williams (2009) mentioned this behavior as men are more concerned about the cost of reducing aflatoxin levels, whereas women exhibit greater awareness when cost was related to the benefits from control. Age might imply greater experience or authority but this factor was not correlated with farmers level of knowledge, awareness, and willingness to pay in this case study. Further, the analysis reveals that marital status is negatively correlated with willingness to pay for aflatoxin control measures. The result can imply that single farmers might have lower liquidity constraints in the household, thus may be more likely to pay for aflatoxin control. However, this result contradicts those of Langiano et al. (2012) who reported that high‐risk behavior of improper safety rules was influenced more by single status than by being married.

Furthermore, our results clearly depicted that education was an important mode of dispersing information and knowledge to the public. Midega et al. (2016) too reported positive and significant effects of education on farmers’ knowledge of maize pests in western Kenya. Strosnider et al. (2006) indicated that education and awareness are crucial factors in alleviating the problems of aflatoxin in developing countries. Many findings reported that income is one of the most salient factors that influence farmers’ perception and awareness (Marmot, 2002; Sabran et al. (2012); Shankardass, Lofters, Kirts, & Quinonez, 2012). In this study, a positive and significant relationship was observed between annual income and knowledge as well as awareness about aflatoxin contamination. This result suggest that poverty and lack of sufficient income might contribute to low levels of awareness and knowledge about aflatoxin, leading to high aflatoxin exposure (Leroy, Wang, & Jones, 2015). This result might also be related to farmers’ reluctance to invest their limited income to learn about and pay for aflatoxin control, especially because the negative effects of contamination are not widely known in the country. However, the positive correlation agrees with the studies reported by Jolly et al. (2009) and Sabran et al. (2012) that people with high income are likely to be more careful about food and are more willing to pay for food safety than those with lower incomes.

Regarding the willingness to pay for aflatoxin control interventions, Tiongco, de Groote, Saak, Narrod, and Scott (2011); Tiongco, Ndjeunga et al. (2011) reported that willingness to pay for risk‐reducing technologies is high for producers who have more assets. In addition, they also found that respondents in Kenya who have had experience with aflatoxicosis outbreaks have high willingness to pay for improved seeds as well as for tarpaulins and metal silos for drying and storing grain. Contact with extension agents, access to credit and prior experience in using a technology play determine the amount a farmer is willing to pay for the technology as has been found for the biological control product Aflasafe in Nigeria (Ayedun et al., 2017). Aflatoxins are colorless, odorless, and invisible; thus, contaminated foods may be perceived as safe and edible (Rodrigues, Venancio, & Lima, 2012). Therefore, farmers with low resource base or low experience in using aflatoxin‐reducing technologies choose not to invest their time, energy, and resources in control interventions as there is no quality grading or price differential for aflatoxin‐free products sold in the markets.

Finally, our study also uncovered some misconceptions about aflatoxin contamination. For example, most farmers are aware that aflatoxin contamination can reduce the price of crops. However, most of them were not aware of the risk of contamination to human health. In addition, although farmers value the importance of good farming practices for aflatoxin prevention, they do not seem to equally appreciate the potential of postharvest management practices for minimizing contamination. Most farmers seem to erroneously thought that preharvest interventions such as seed treatments with chemicals is more effective than postharvest practices such as the use of appropriate storage practices. In this regard, the introduction of nonchemical storage options, such as the Purdue Improved Crop storage (PICS) bags, might play an important role in reducing contamination. PICS makes the use of chemicals unnecessary and allows the farmer to store grains until needed for consumption or sale.

## 5 CONCLUSIONS

This is the first report on the incidence of aflatoxin contamination in crop products produced by farmers for household consumption in Eastern DRC. All samples were aflatoxin positive and 68% contained total aflatoxin levels over the EU maximum tolerable level of 4 μg/kg. The processed samples were more contaminated than unprocessed samples. The results from this exploratory study also showed that farmers’ knowledge and awareness about aflatoxins were mostly influenced by the level of income and education, while marital status, and sex of the household head were negatively correlated with willingness to pay for control measures. To reduce the exposure and negative impact of contamination from farm to consumer, it is critical that farmers in Eastern DRC receive training on the causes, control, and consequences of aflatoxins and put knowledge into action. Increasing knowledge among farmers might result in creating greater awareness and understanding of the aflatoxin problem. Any attempts to change preharvest and postharvest management practices will need interventions that smallholder farmers will accept, adopt, and maintain. At the subsistence farm and processing levels, application of biological control in conjunction with good agricultural and storage practices as well as efficient postharvest management should be introduced to farmers through training as part of efforts to reduce the risk of aflatoxin contamination. As this study was specifically focused toward farmers, the finding did not represent the knowledge and awareness level of the public at large. Thus, government bodies, private organizations, and nongovernmental organizations, as well as national media networks need to play a prominent role in raising awareness of the public health impacts of aflatoxin. Considering the necessity of achieving food security and food safety for vulnerable people in these areas, there is also a need for data and risk management capacity tools for locally driven policy reform. In a market‐based system, support for institutional innovations to encourage private sector investments for aflatoxin reduction is more likely to be successful. With such concerted interventions, farmers will continue to increase the necessary incentives and capacity to respond adequately to aflatoxin control. Nevertheless, a single factor alone might not be enough to explain the behavior of farmers. The role of other socio‐demographic and socioeconomic factors, such as farm size, social participation, extension services, market orientation, and economic motivation, needs to be explored further to obtain a better understanding of farmers’ knowledge and awareness on aflatoxins.

## ETHICAL STATEMENT

This study does not involve any human or animal testing.

## CONFLICT OF INTEREST

The authors have no conflict of interests.
